# Case Report: Multi-drug-resistant pneumonia and chronic enteropathy in a Bernadoodle with selective IgA deficiency

**DOI:** 10.3389/fvets.2026.1830827

**Published:** 2026-07-15

**Authors:** Marselle Kovarsky, Jean Rifkin, Khelsea Bahr

**Affiliations:** VCA Animal Hospitals, Los Angeles, CA, United States

**Keywords:** canine infectious respiratory disease complex, case report, immunoglobulin a, multidrug resistance, primary immunodeficiency

## Abstract

This case is the first to describe a selective immunoglobulin A deficiency (sIgAD) in a Bernadoodle with recurrent multi-drug-resistant (MDR) pneumonia and a chronic enteropathy. The patient experienced chronic coughing and regurgitation from puppyhood, and advanced imaging and endoscopic procedures documented MDR *Bordetella bronchisceptica* (Bb) and Mycoplasma infections. Serum immunology testing documented sIgAD. Clinical signs have been well-managed with long-term prophylactic antibiotics, anti-inflammatory steroids, probiotics, and hydrolyzed protein diet. Low serum IgA levels have not been previously documented in the Bernese Mountain dog nor designer cross breeds, such as the Bernadoodle.

## 1 Introduction

This report is the first to describe sIgAD in a Bernadoodle with recurrent MDR Bb and Mycoplasma respiratory infections and a chronic enteropathy. IgA is one of the most important molecules in maintaining mucosal barrier homeostasis in the respiratory and gastrointestinal tract. In dogs, serum IgA does not reach full adult levels until 12 months of age (1). One study evaluated serum immunoglobulin levels in 10-month-old Beagles and found an age-dependent increase in serum IgA levels between 0.8 and 1.6 years (2). Additionally, in German shepherds, serum IgA levels increased from 7 weeks to 13 months and then stabilized between 13–18 months (3). IgA concentrations vary widely amongst breeds, but sex and altered status do not affect levels (4).

In people, sIgAD has been well-described and is the most common primary immunodeficiency. It is immunologically defined by a serum IgA concentration < 0.07 g/L, with normal concentrations of IgG and IgM. Most people diagnosed with sIgAD are asymptomatic, likely due to compensation from other immunoglobulins, especially IgM. However, infectious diseases, primarily of the respiratory and gastrointestinal tracts, are more common in people with sIgAD compared to people with normal IgA levels. Mild or moderate recurrent upper respiratory infections of viral or bacterial origin typically begin in early childhood. These individuals have an increased risk of allergies, auto-immune disease, and several gastrointestinal disorders (5, 6).

SIgAD is also thought to be the most common canine immunodeficiency. Breeds that have been documented to have sIgAD or low serum IgA levels include the Beagle, Shar-Pei, German shepherd, Hovawart, Norwegian elkhound, Nova Scotia duck tolling retriever, Bullterrier, Golden retriever, Labrador retriever, and Irish setter (4, 7, 8). Previous cut-offs for IgA deficiency in dogs have been established at 0.15–0.18 g/L (15–18 mg/dL) for purebred dogs, and 0.22 g/L (22 mg/dL) for crossbred dogs (7, 9). Breed-specific serum IgA ranges for dogs <12 months old have not been established (1).

SIgAD in dogs is characterized by low concentrations of serum IgA, normal concentrations of IgG and IgM, normal T-cell function, the presence of autoantibodies, and a defect in the maturation or terminal differentiation of IgA B cells into IgA-secreting plasma cells. Prior case studies in dogs with sIgAD have noted mild recurrent respiratory infections (previously associated with Bb and canine parainfluenza virus), dermatitis, chronic gastrointestinal signs, and seizures (7).

## 2 Case description

An intact male Bernadoodle was noted to have reverse sneezing, sneezing, and coughing since being acquired from the breeder at 8-weeks-old. Treatment trials by the referring veterinarian included dextromethorphan/guaifenesin (0.78 mg/kg/7.8 mg/kg PO q8hr), doxycycline (6.5 mg/kg PO q12hr), amoxicillin/clavulanate (15 mg/kg PO q12hr), trimeprazine/prednisolone (0.65 mg/kg / 0.25 mg/kg PO q12hr tapering dose), maropitant (2.2 mg/kg PO q24hr), and Proviable-DC [Nutramax Laboratories Veterinary Sciences, Lancaster, SC]. Coughing persisted despite all treatment trials. At 12-weeks-old, he was vaccinated with intranasal Bordetella vaccine [Vanguard B Intranasal, Zoetis, Parsippany, NJ], and routine fecal testing was positive for Giardia [SNAP Giardia antigen test, IDEXX Laboratories Inc., Westbrook, ME], which was treated with metronidazole (15 mg/kg PO q12hr) and fenbendazole (50 mg/kg PO q24hr). An upper respiratory PCR panel [IDEXX] was positive for Bb at 13-weeks and 15-weeks old.

At 6-months-old, he was presented to specialty referral hospital for evaluation of his chronic respiratory signs and Bb infection. A recheck upper respiratory PCR panel [IDEXX] remained positive for Bb. Azithromycin (9.1 mg/kg PO q24hr) and hydrocodone (0.18 mg/kg PO q12hr) were prescribed for 10 days, which resolved the coughing.

At 8-months-old, the coughing returned. Head and thoracic computed tomography noted mild nasal turbinate atrophy and moderate lymphadenopathy (bilateral mandibular, retropharyngeal, superficial cervical, cranial mediastinal), with no significant pulmonary abnormalities. Bronchoscopy noted a small amount of mucus with patchy hyperemia in the trachea and lower airways. Bronchoalveolar lavage cytology noted marked neutrophilic inflammation, and culture was positive for MDR Bb [Antech Diagnostics, Irvine, CA] and Mycoplasma species [Colorado State University (CSU) Veterinary Diagnostic Laboratory, Fort Collins, CO] (Table 1). Upper respiratory PCR panel [IDEXX] was positive for Bb and *Mycoplasma cynos* (Mc). Following the procedure, he developed lethargy and fever. Thoracic radiographs noted a patchy interstitial to alveolar pattern in the right middle and caudal subsegment of the left cranial lung lobe, consistent with aspiration pneumonia. He was hospitalized on ampicillin/sulbactam (30 mg/kg IV q8hr) and discharged on azithromycin (8.7 mg/kg PO q24hr).

**Table 1:** Susceptibility table for MDR Bb obtained from bronchoalveolar lavage.

Minimal Inhib. Conc. Organism #1			
Test	MIC (ug/ml)	Interpretation	Suggested dose
Amikacin	<=8	S	15 mg/kg IV or SC q24h
Clavamox	<=2	S	13.75 mg/kg PO q12
Enrofloxacin	<=0.5	S	5 mg/kg PO q24
Gentamicin	<=2	S	6 mg/kg IV or SC q24h
Marbofloxacin	<=1	S	2.75 mg/kg PO q24h
Amoxicillin	N/A	R	
Ampicillin	N/A	R	
Cefadroxil	> = 8	R	
Cefazolin	> = 8	R	
Cefovecin	> = 8	R	
Cefoxitin	> = 32	R	
Cefpodoxime	> = 8	R	
Ceftiofur	> = 8	R	
Cephalexin	> = 8	R	
Potentiated sulfonamide	> = 1	R	
Bordetella add on sensitivities			
Test	Results	Adult reference interval	Status
Azithromycin	S		
Doxycycline	S		

One week after discharge, he was noted to have regurgitation and progressive coughing. Recheck thoracic radiographs noted a progressive interstitial to alveolar pattern in the ventral lungs. Antibiotic therapy was switched from azithromycin to amoxicillin/clavulanate (17.3 mg/kg PO q12hr).

At 8.5-months-old, he continued to have coughing, lethargy, anorexia, and fever. Due to persistent intermittent regurgitation, further gastrointestinal work up was performed. A baseline cortisol was normal [3.4 (1–5 ug/dL)] [Antech]. Abdominal ultrasound was unremarkable for his age. Gastrointestinal panel [Texas A&M University Gastrointestinal Laboratory, College Station, TX] noted low-normal cobalamin [349 (251–908 ng/L)], normal folate [17.7 (7.7–24.4 mg/L)], and suboptimal serum trypsin-like immunoreactivity [9 (10.9–50 ug/L)]. A fecal keyscreen PCR [Antech] was positive for Giardia duodenalis and Cystoisospora spp. He was hospitalized on ampicillin/sulbactam (30 mg/kg IV q8hr) and enrofloxacin (5.2 mg/kg IV q24hr) and discharged on amoxicillin/clavulanate (17.6 mg/kg PO q12hr), marbofloxacin (3.5 mg/kg PO q24hr), prednisone (0.52 mg/kg q24hr x 10 days), sucralfate, omeprazole, maropitant, probiotics, cyanocobalamin, praziquantel/pyrantel pamoate/febantel, and a hydrolyzed protein diet. Coughing improved and recheck radiographs two weeks later noted mildly improved but persistent patchy interstitial to alveolar pattern in the right middle and left cranial lung lobes. Antibiotic therapy with amoxicillin/clavulanate and marbofloxacin was continued.

At 9.5-months-old, he developed lethargy, anorexia, green nasal discharge, and fever. Repeat thoracic radiographs noted a severe ventral alveolar pattern. Endotracheal wash was performed, and cytology noted marked neutrophilic inflammation with extracellular cocci and suspected intracellular bacteria. Culture grew a MDR Bb [Antech] and Mycoplasma species [CSU] (Table 2). Antibiotic therapy was switched to amikacin (15 mg/kg SQ q24hr), but due to persistent fever, coughing, and green nasal discharge, it was switched again one week later to meropenem (12 mg/kg SQ q12hr).

**Table 2:** Susceptibility table for MDR Bb obtained from endotracheal wash.

Minimal Inhib. Conc. Organism #1			
Test	MIC (UG/ML)	Interpretation	Suggested dose
Amikacin	<=8	S	15 mg/kg IV or SC q24h
Clavamox	<=2	S	13.75 mg/kg PO q12
Gentamicin	<=2	S	6 mg/kg IV or SC q24h
Marbofloxacin	<=1	S	2.75 mg/kg PO q24h
Enrofloxacin	1	I	10 mg/kg PO q12h
Amoxicillin	N/A	R	
Ampicillin	N/A	R	
Cefadroxil	> = 8	R	
Cefazolin	> = 8	R	
Cefovecin	> = 8	R	
Cefoxitin	> = 32	R	
Cefpodoxime	> = 8	R	
Ceftiofur	> = 8	R	
Cephalexin	> = 8	R	
Potentiated sulfonamide	> = 1	R	
Bordetella add on sensitivities			
Test	Results	Adult reference interval	Status
Azithromycin	I		
Doxycycline	I		
Special sensitivities			
Test	Results	Adult reference interval	Status
Special sensitivities	03/13/2025		
	Comment: tested sensitive to meropenem		

At 10.5-months-old, he was energetic and afebrile but had a persistent cough; recheck radiographs noted an improved but persistent ventral alveolar pattern. Gentamicin (4 mL undiluted gentamicin 5% nebulized over 10 min q12hr) was started in addition to meropenem.

At 11.5-months-old, coughing worsened once discontinuing antibiotics. A repeat upper respiratory PCR panel [IDEXX] remained positive for Bb and Mc, and culture from the upper respiratory swab noted a MDR Bb [IDEXX] (Table 3). Azithromycin (7.2 mg/kg PO q24hr) and prednisone (0.14 mg/kg PO q48hr) were prescribed. Due to chronic respiratory and gastrointestinal signs, serum immunology testing was performed. Results noted a selective IgA deficiency: IgA < 26 [35–270 mg/dL], IgG 545 [670–1,650 mg/dL], IgM 346 [100–400 mg/dL] [Animal Health Diagnostic Center, Ithaca, NY].

**Table 3:** Susceptibility table for MDR Bb obtained from upper respiratory swab.

Minimal Inhib. Conc. Organism #1			
Test	MIC (UG/ML)	Interpretation	Suggested dose
Amikacin	<=8	S	15 mg/kg IV or SC q24h
Enrofloxacin	<=0.5	S	5 mg/kg PO q24
Gentamicin	<=2	S	6 mg/kg IV or SC q24h
Marbofloxacin	<=1	S	2.75 mg/kg PO q24h
Amoxicillin	N/A	R	
Ampicillin	N/A	R	
Cefadroxil	> = 8	R	
Cefazolin	> = 8	R	
Cefovecin	> = 8	R	
Cefoxitin	> = 32	R	
Cefpodoxime	> = 8	R	
Ceftiofur	> = 8	R	
Cephalexin	> = 8	R	
Clavamox	N/A	R	
Potentiated sulfonamide	> = 1	R	
Bordetella add on sensitivities			
Test	Results	Adult reference interval	Status
Azithromycin	S		
Doxycycline	S		

Azithromycin was tapered to q48hr dosing after several months of therapy. During the most recent recheck at 2-years-old, the patient remains playful with minimal coughing. Currently he remains on azithromycin q48hr and prednisone q48hrs long-term for cough management, in addition to probiotics and a hydrolyzed protein diet for regurgitation management.

## 3 Discussion

This patient had recurrent respiratory infections with MDR Bb and Mycoplasma. Differential diagnoses included sIgAD, intermittent aspiration, ciliary dyskinesia, and chronic bronchitis. Sperm evaluation was not performed in this patient to evaluate for ciliary dyskinesia.

Bb is one of the major pathogenic bacteria for canine infectious respiratory disease complex (CIRDC), which is a worldwide epidemic involving multiple viral and bacterial pathogens. Mycoplasma species are present in the upper and lower respiratory tract of healthy and symptomatic dogs, making its role in CIRDC more complicated. *M. canis*, *M. cynos*, *M. edwardii*, and *M. spumans* have been isolated from the lower respiratory tract of dogs with CIRDC, and *M. cynos* was found to have a primary pathogenic role in both lower and upper respiratory tract disease in dogs (10).

This patient also had evidence of a chronic enteropathy characterized by chronic regurgitation, low-normal serum cobalamin levels, and recurrent Giardiasis. SIgAD and chronic enteropathies have been previously well documented in German shepherds (11). An association between SIgAD and several gastrointestinal disorders, including celiac disease, Crohn’s disease, ulcerative colitis, chronic or recurrent *Giardia lamblia* infections, and gastric and colonic adenocarcinoma have been reported in people (6). This patient also had a subnormal serum trypsin-like immunoreactivity level; however, based on the updated reference intervals and decision thresholds listed by the Gastrointestinal Laboratory at Texas A&M, exocrine pancreatic insufficiency (EPI) is unlikely. Additionally, this patient’s gastrointestinal signs were well controlled without pancreatic enzyme therapy, further making EPI unlikely.

There is currently no specific treatment for sIgAD in people. Antimicrobial therapy for upper and lower respiratory tract infections should be based on culture and sensitivity (6). In this patient, antibiotics were chosen based on susceptibility profiles when available. The administration of antimicrobials is one of the most effective ways to control Bb and Mycoplasma, but the emergence of MDR bacteria may lead to clinical failures (12). Antibiotic susceptibility profiles of Bb have historically shown low levels of resistance (<9% resistance); however, in a recent review of Bb in China, 100% of isolates evaluated were MDR (13, 14). Resistance to ampicillin, trimethoprim/sulfamethoxazole, cefovecin, and doxycycline has been documented (13, 15). Mycoplasma species lack a cell wall, and therefore are inherently resistant to penicillins. Mycoplasma resistance to macrolides, fluoroquinolones, and tetracyclines has been documented (16).

There is conflicting evidence regarding optimal duration of treatment for canine upper and lower respiratory tract infections. Historical studies recommend treatment to be continued 1–2 weeks beyond radiographic resolution of pneumonia (17). However, radiographic resolution of pneumonia may be misleading, as image normalization lags clinical improvement. Additionally, Mc can persist in the lungs for up to 3 weeks after infection, and some Mycoplasma species can form a biofilm that can allow them to persist longer (18, 19). More recent studies recommend a 10-day course of antibiotics for uncomplicated pneumonia, or continuing antibiotics until clinical improvement and serum C-reactive protein (sCrp) normalizes (18, 20). Elevated sCrp levels have been documented in human patients with sIgAD, but to the authors’ knowledge, has not been specifically evaluated in dogs with sIgAD (21).

In this patient, antibiotic duration ranged greatly, and he frequently continued to have clinical signs and radiographic evidence of pneumonia despite concurrent antimicrobial therapy. At the time of writing, the patient has remained on azithromycin for >1 year. In humans with sIgAD with recurrent respiratory infections, daily prophylactic antibiotics can be considered (6, 22). It is unknown if monitoring sCrp in this patient would have been beneficial in guiding duration of antimicrobial therapy.

Vaccines for Bb have been commonly used in dogs since their development in the 1970s/1980s. Both injectable and intranasal vaccines are available in dogs. The intranasal administration of avirulent live cultures of Bb initiates production of local secretory IgA antibodies and systemic IgG antibodies. Secretory IgA antibodies are detected in dogs within 4 days of vaccination (23). Humans with sIgAD have poor mucosal response to vaccines (24). The mucosal response in dogs with sIgAD remains open, and to the authors’ knowledge has not been specifically evaluated.

Bb vaccine strains have been reported to survive for at least two weeks after immunization with live attenuated vaccines (25). This patient received a live attenuated intranasal vaccine at 3-months-old. The first upper respiratory PCR panel was performed 1-week post vaccination, so a false positive result was possible from vaccine interference. The authors suspect that the upper respiratory PCR panels performed 12-weeks and further post-vaccination were less likely to be affected by vaccine interference, and more likely to represent true infection.

Virulence reactivation (from avirulent to virulent) is a low possibility, but vaccine-derived Bb has been reported to be transmissible from dogs to immunocompromised people (26). Humans with sIgAD should avoid live attenuated vaccines, and some vaccines are contraindicated (specifically oral polio vaccine, Bacille Calmette-Guerin, and yellow fever) due to the risk for developing disseminated infections (6). Since this patient had upper respiratory signs prior to Bb vaccination, the authors speculate that a vaccine-derived Bb infection was unlikely but cannot be fully excluded. A limitation of this case was that Bb testing was not performed prior to vaccination.

Future treatment considerations for dogs with sIgAD may include immunoglobulin therapy and sublingual immunotherapy. Immunoglobulin replacement therapy is controversial in people with sIgAD, as clinical efficacy is minimal. Very rarely patients with sIgAD have had anaphylaxis after administration of intravenous immunoglobulin (IVIG), thought to be due to high levels of anti-IgA antibodies in the recipient and small amounts of IgA in IVIG. Subcutaneous IgG infusions have been tolerated without reaction in patients that previously had an anaphylaxis reaction to IVIG (27). Studies in mice have noted that local administration of serum IgA can be protective against certain infections, so the intranasal and/or oral administration of serum IgA is a potential tract to be explored in human and veterinary medicine (5). More recent research also suggests that long-term administration of sublingual immunotherapy with inactive bacteria extract, similar to allergen immunotherapy, may be an effective approach for prophylaxis against respiratory tract infections in people with sIgAD (28).

While low serum IgA levels have been previously documented in Standard Poodles and adult crossbreed dogs from local pounds, low serum IgA levels have not been previously documented in the Bernese Mountain Dog, nor designer cross breeds, such as the Bernadoodle (4, 9). With the widespread popularity of designer cross breeds, future studies could characterize IgA cutoffs for these designer breeds.

An additional limitation of our study was that immunology testing was performed at 11.5-months-old. Serum IgA is thought to stabilize at 12-months-old, and reference ranges have not been established for dogs <12 months old. As such, a healthy, age-matched, designer cross breed dog was concurrently tested and used as a control.

## Data Availability

The original contributions presented in the study are included in the article/supplementary material, further inquiries can be directed to the corresponding author.
